# Thermal and electrical properties of PVDF modified Co_3_O_4_ functionalized MWCNTs

**DOI:** 10.1039/d4ra07239a

**Published:** 2025-03-24

**Authors:** Zia Ur Rehman, Farhan, Shabir Ahmad, Hameed Ullah, Sara A. Alqarni, Shanshan Yao, Khalid Ali Khan, Magdi E. A. Zaki

**Affiliations:** a Institute for Advanced Materials, College of Materials Science and Engineering, Jiangsu University Zhenjiang 212013 P. R. China ziamarwat@hu.edu.pk ziamwt1@gmail.com; b Department of Chemistry, Islamia College University Peshawar-25120 Khyber Pakhtunkhwa Pakistan; c Department of Chemistry, Hazara University Mansehra-21120 Khyber Pakhtunkhwa Pakistan; d Department of Chemistry, College of Science, University of Jeddah Jeddah Saudi Arabia; e Applied College, Center of Bee Research and its Products (CBRP), Unit of Bee Research and Honey Production, King Khalid University P.O. Box 9004 Abha 61413 Saudi Arabia; f Department of Chemistry, College of Science, Imam Mohammad Ibn Saud Islamic University Riyadh 11623 Saudi Arabia

## Abstract

This research examines the synthesis of Co_3_O_4_–MWCNTs nano-hybrid structures and their incorporation into PVDF polymer nanocomposite thin films *via* the solution casting method. The study comprehensively characterizes the structural, thermal, and electrical properties of the resulting nanocomposites using techniques such as SEM, XRD, FTIR, TGA, TDA, DSC, and impedance spectroscopy. XRD confirmed the crystalline structure and phase transition of the PVDF/Co_3_O_4_–MWCNTs nanocomposites, while FTIR analysis revealed the presence of α- and β-phases of PVDF. TGA, TDA, and DSC results revealed enhanced thermal stability, highlighting the potential for high-temperature applications. Notably, the dielectric properties significantly improved at 0.5 wt% Co_3_O_4_ and 0.3 wt% MWCNTs. The electrical conductivity of the nanocomposites increased with higher nano-hybrid content, owing to strong interactions between the PVDF polymer and nano-fillers. This work provides insight into the development of advanced nanocomposites with superior thermal and electrical properties, which could be used in electronic and energy storage devices. The novelty of this study lies in the effective combination of Co_3_O_4_ and MWCNTs to enhance the properties of PVDF, offering a promising material for future industrial applications.

## Abstract 2

This research examines the synthesis of Co_3_O_4_–MWCNTs nano-hybrid structures and their incorporation into PVDF polymer for electrical and thermal properties.

## Graphical abstract

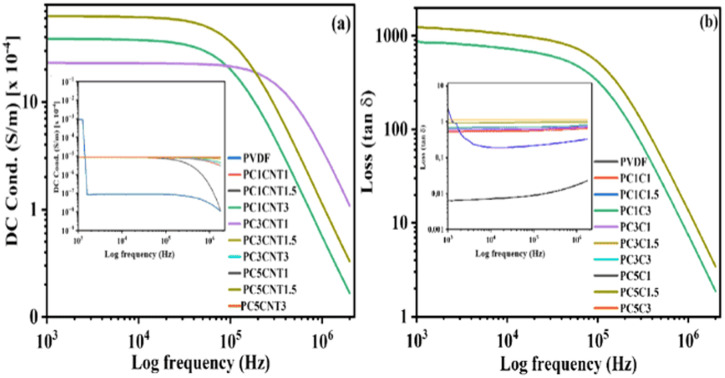

## 1 Introduction

Polymer nanocomposites are the result of addition of organic or inorganic nano-fillers into the polymers.^1,2^ Polymer nanocomposites are a new class of macromolecules that have received significant attention over pure polymers due to their enhanced physical properties, such as thermal, mechanical, and electrical performance.^3–5^ The physical characteristics of polymers are further improved by using many functional nanomaterials which are employed as reinforcements in polymer nanocomposites, particularly functionalized carbon nanotubes^6–8^ which impart specific characteristics like high aspect ratio, high mechanical strength, unique thermal stability/decomposition and electrical properties to the polymer nanocomposites to get ideal nano-materials with significantly enhanced properties such as toughness, solvent resistance, optical properties, and electrical and thermal conductivity.^9–11^ The characteristics of the nanomaterials, *i.e.*, the chemical, mechanical, thermal and electrical properties could be significantly improved upon fabrication of hybrid membranes.^12–14^ Organic/inorganic hybrid nanocomposites are becoming more important in the present time as thin polymer membranes. The unusual versatility of the hybrid nanomaterials is largely dependent upon the selection of polymers and fillers available to researchers.^15–18^

Poly (vinylidene fluoride) (PVDF) is considered a well-known material for adoption in piezoelectric and pyroelectric materials.^19–21^ From a scientific point of view, PVDF is one of the most studied polymeric materials, mostly used in the fields of storage devices such as capacitors, water purifying devices, microwave transducers, sensors, and energy harvesting systems.^22–24^ In pure form, PVDF has poor thermal and electrical properties and the improvement in these properties and piezoelectricity is still challenging.^20,25^ The addition of nano-fillers to PVDF enhances the piezo and pyroelectric performance.^26^ This behavior is mainly contributed by the polar β-phase, rather than the α-phase, as the polar β-phase is the functional phase which imparts the highest dipole moment resulting in high piezoelectricity.^27,28^ One-dimensional (1D) nanostructures in the form of fibers, wires, tubes, and rings have attracted a lot of attention due to their infinite applications in electronics, catalysis, drug delivery, and antibacterial and antioxidant properties.^29,30^ Previously, various dimensional nanostructures of Co_3_O_4_ have been synthesized, such as nanoparticles,^31^ nanofibers,^32,33^ nanowires and nanotubes.^34–37^ Among these nanostructures, 1D cobalt oxides (Co_3_O_4_) have received much attention and growing interest.^38–40^ 1D Co_3_O_4_ is technologically a very important metal oxide due to its applications in catalysts, as anode materials in lithium-ion batteries, sensors, and electrical and electronic devices.^37,41–43^ Multiwall carbon nanotubes (MWCNTs) are also very attractive materials due to their high specific area, chemical resistance, high mechanical strength, and unique electrical and thermal properties.^11,44,45^ It will be interesting to study the combined properties of Co_3_O_4_–MWCNTs blend with PVDF.

In the present study, we try to see the impact of the combined effect of Co_3_O_4_–MWCNTs nanostructures on the crystallinity and thermal and electrical behaviors of PVDF.

## 2 Experimental

### 2.1 Materials

The powdered form of poly (vinylidene fluoride) (*M*_w_ ∼ 534 000 g mol^−1^), polyvinylpyrrolidone (PVP) (*M*_w_ – 1 300 000 g mol^−1^), tetrahydrofuran (THF), nitric acid (HNO_3_), sulphuric acid (H_2_SO_4_), cobalt nitrate hexahydrate [Co(No_3_)]. Multiwall carbon nanotubes (MWCNTs) were utilized after its functionalization as reported.^46^ The MWCNTs were purified by calcination at 500 °C for 15 minutes to eliminate the impurities such as metals and amorphous carbon. The rest of the chemicals used in the fabrication of nanocomposites were consumed without subjecting to additional purification protocols.

### 2.2 Synthesis of 1D Co_3_O_4_ nanostructure

The one-dimensional (1D) nanostructures of Co_3_O_4_ were synthesized using an electrospinning technique as described by.^38^ Cobalt nitrate hexahydrate (Co(NO_3_)_2_·6H_2_O, 1.5 g) was dissolved in 10 mL of tetrahydrofuran (THF) under constant stirring for 1 hour to form a cobalt precursor solution. Polyvinylpyrrolidone (PVP, 1.2 g) was added to the solution and stirred continuously for 5 hours to form a homogeneous PVP–cobalt solution. The electrospinning setup consisted of a 5 mL syringe filled with the solution, which was connected to a syringe pump. The syringe pump was linked to a high-voltage power supply and a stainless-steel needle. The needle was positioned 15 cm from a collector surface covered with aluminum foil. A voltage of 15 kV was applied to initiate the electrospinning process. Nanowires of Co_3_O_4_ were collected on the surface of the collector as the solution was electrospun into fibers. After electrospinning, the collected nanowires were carefully scraped off using a spatula and stored in a glass vial for further processing. To remove the PVP, the nanowires were calcined in a furnace at 600 °C for 2 hours, allowing the formation of Co_3_O_4_ nanostructures. The calcination process was conducted under air atmosphere with a ramp rate of 5 °C min^−1^.

### 2.3 Procedure for MWCNTs functionalization

MWCNTs were functionalized by the following standard protocol as reported.^46^

### 2.4 Synthesis of Co_3_O_4_–MWCNTs/PVDF nanocomposites membranes

Two separate solutions were prepared to synthesize Co_3_O_4_–MWCNTs/PVDF nanocomposite membranes. The Co_3_O_4_–MWCNTs solution was prepared by dispersing a specified amount of Co_3_O_4_–MWCNTs in 10 mL of tetrahydrofuran (THF) and subjecting the mixture to sonication for 2 hours. Simultaneously, the PVDF dispersion was prepared by dissolving the required amount of polyvinylidene fluoride (PVDF) in THF, followed by constant stirring for 2 hours at room temperature. The two prepared solutions were then combined and subjected to further sonication for 3 hours to ensure a uniform dispersion. After sonication, the mixture was refluxed at a constant temperature of 70 °C for 7 hours to promote better integration of the Co_3_O_4_–MWCNTs nanostructures into the PVDF polymer. The dispersion was then sonicated for an additional 3 hours to achieve an even better distribution of the nanostructures. The resulting nanocomposite mixture was carefully poured into a Petri dish and placed in an oven at 70 °C for 6 hours to completely remove the solvent. The Co_3_O_4_–MWCNTs/PVDF nanocomposite membrane was then obtained as the final product.

For the synthesis of nanocomposites with varying MWCNT content, functionalized multi-walled carbon nanotubes (MWCNTs) were added in three different weight percentages (wt%) of 0.1, 0.15, and 0.3 wt% relative to the total polymer weight. Similarly, 1D Co_3_O_4_ nanowires were also added in weight percent of 0.1 wt%, 0.3 wt% and 0.5 wt% with keeping constant quantity of PVDF polymer. Total ten nanocomposites membranes were prepared *i.e.* PVDF blank film, PC1CNT1 (0.1 wt% Co_3_O_4_ + 0.1 wt% CNTs), PC1CNT1.5 (0.1 wt% Co_3_O_4_ + 0.15 wt% CNTs), PC1CNT3 (0.1 wt% Co_3_O_4_ + 0.3 wt% CNTs), PC3CNT1 (0.3 wt% Co_3_O_4_ + 0.1 wt% CNTs), PC3CNT1.5 (0.3 wt% Co_3_O_4_ + 0.15 wt% CNTs), PC3CNT3 (0.3 wt% Co_3_O_4_ + 0.3 wt% CNTs), PC5CNT1 (0.5 wt% Co_3_O_4_ + 0.1 wt% CNTs), PC1CNT1.5 (0.5 wt% Co_3_O_4_ + 0.15 wt% CNTs) and PC5CNT3 (0.5 wt% Co_3_O_4_ + 0.1 wt% CNTs). A detailed description of all the PVDF nanocomposites membranes has been given in Table 1.

**Table 1:** Details description of Co_3_O_4_ and MWCNTs wt% in the resulted nanocomposites

Code	PVDF (wt%)	Co_2_O_4_ (wt%)	MWCNTs (wt%)
PVDF	100	0.0	0.0
PC1CNT1	99.8	0.1	0.1
PC1CNT1.5	99.75	0.1	0.15
PC1CNT3	99.6	0.1	0.3
PC3CNT1	99.6	0.3	0.1
PC3CNT1.5	99.55	0.3	0.15
PC3CNT3	99.4	0.3	0.3
PC5CNT1	99.4	0.5	0.1
PC5CNT1.5	99.35	0.5	0.15
PC5CNT3	99.2	0.5	0.3

### 2.5 Characterization of the Co_3_O_4_ and PVDF/Co_3_O_4_–MWCNTs nanocomposites

The nanostructures of Co_3_O_4_ were characterized by various physical techniques such as transmission electron microscope (TEM), X-rays diffraction (XRD), Fourier transformed infrared (FTIR), and electrospinning. While the PVDF nanocomposites films containing hybrid Co_3_O_4_–MWCNTs nanostructures were characterized and analyzed by FTIR, XRD, Thermo-gravimetric analysis (TGA), Thermal differential analysis (TDA), Differential scanning calorimetry (DSC) and Direct current (DC) conductivity. Xpert pro. of Cu-based X-rays source which produce radiation of Kα type (*λ*: 1.542 Å). XRD pattern was used for crystal analysis of nanocomposite films. FTIR spectrometer in wavelength range of 4000–400 cm^−1^ was used to achieve FTIR spectra of the resulting nanocomposite films. The TEM analyzer (Model-JSM 6490) was used to study and analyzed the 2D structural morphology of Co_3_O_4_ nanowires. The TGA analyzer (Model-TGA 7) was carried out in a heating range of about 20–800 °C at heating rate of 5 °C min^−1^ using nitrogen environment. The TDA data was obtained from TGA data using differential technique. DSC analyzer was carried out in temperature range 0–200 °C, at heating rate of ∼10 °C min^−1^ was used to obtain the DSC data. The electrical properties of the PVDF-nanocomposites were measured by using inductance-*L*, capacitance-*C*, and resistance-*R* (LCR) meter. The data of electrical conductivity of the films were obtained by applying silver (Ag) paste on both sides of the films. The silver (Ag) paste was achieved as a result of well mixing of silver-metal in isoamyl acetate solvent. The prepared silver paste was then applied on the pure PVDF and PVDF/Co_3_O_4_–MWCNTs nanocomposites to measure the conductivity behaviors of the resulting nanocomposite membranes.^47^ The two terminals of the LCR meter were connected to the two ends of the thin films containing silver paste and the program was run to measure the DC-conductance and dielectric loss of the prepared nanocomposite membranes.

## 3 Results and discussion

### 3.1 Analysis of Co_3_O_4_ nanowires

**Fig. 1:**
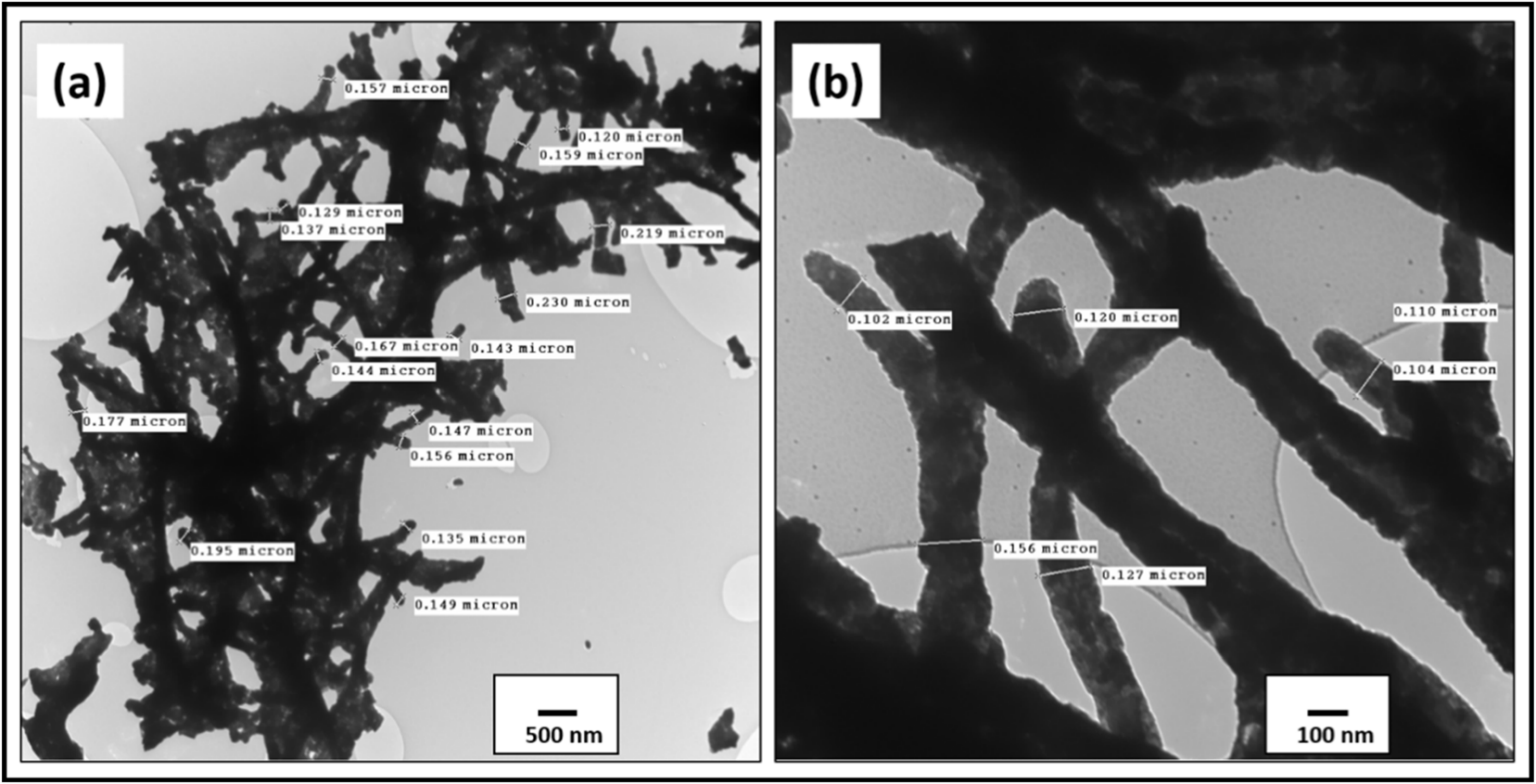
TEM micrograph of cobalt oxide-nanostructures (a) low, and (b) high magnification, respectively.

The surface morphology of the cobalt oxide nanowires was carried out by the TEM microscope which confirmed the formation of one-dimensional Co_3_O_4_ nanostructures illustrated in low and high magnifications, Fig. 1(a) and (b). The external surface of 1D Co_3_O_4_ nanowires looks grainy, compact and also rough at the corner. The rough edges arise during removal of solvent and PVP which result in the grains filling the space and make the surface rough. The length of nanowires exceeded over 1 μm while their diameters range in between 194 nm and 104 nm respectively as labelled in the given TEM micro-images.

**Fig. 2:**
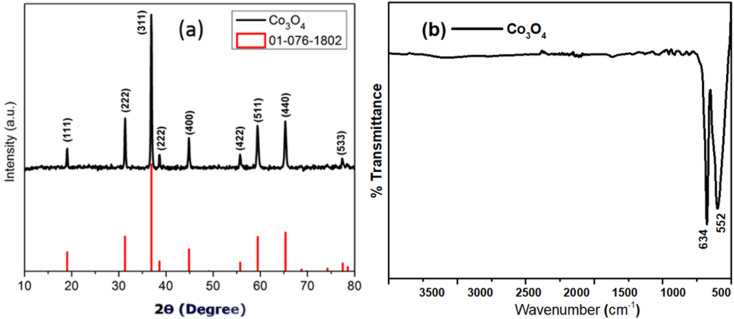
X-rays diffraction pattern (a), and FTIR spectrum (b) of Co_3_O_4_ nanowires.

The electrospun cobalt oxide nanowires obtained after calcination were characterized by powder XRD, FTIR spectroscopy and TEM. The XRD pattern of Co_3_O_4_ as labelled in Fig. 2(a) which was analyzed for phase changes and crystal structure *via* the X'Pert HighScore software. The diffraction pattern of the cobalt oxide nanowires corresponded to Co_3_O_4_ crystalline phase which matched perfectly with the diffraction PDF file No. 01-076-1802. The crystalline phase Co_3_O_4_ is cubic crystal system represented by space group *Fd*_3_*m* and space group number 227 respectively. The reflections of the Co_3_O_4_ XRD pattern appeared at 2*θ* = 19.02°, 31.31°, 36.90°, 38.60, 44.88°, 55.74°, 59.45°, 65.34°, 77.47° and the respective miller indices are (111), (222), (311), (222), (400), (422), (511), (411), and (533), respectively. The XRD pattern showed that there are no impurities in it nor any residues of the PVP due to high temperature and the XRD pattern is solely assigned to Co_3_O_4_ phase.

The FTIR spectrum of 1D Co_3_O_4_ crystalline is given in Fig. 2(b), which confirms the formation of the crystalline Co_3_O_4_ nanowires. The FTIR spectrum show two prominent peaks appearing at different absorption position at 2*θ* of 534 cm^−1^, and 652 cm^−1^. The peak appear at position 534 cm^−1^ is allocated to the strong stretching vibration of Co–O (where Co exist as Co^3+^) occupying the octahedral corners of the corresponding cubic spinel lattice while the absorption peak at 652 cm^−1^ is allocated to the stretching Co–O bond vibration (where Co exist as Co^2+^) occupying the tetrahedral corners of the spinel lattice.^48,49^

### 3.2 Structural analysis of PVDF nanocomposites with Co_3_O_4_–MWCNTs nanostructure

**Fig. 3:**
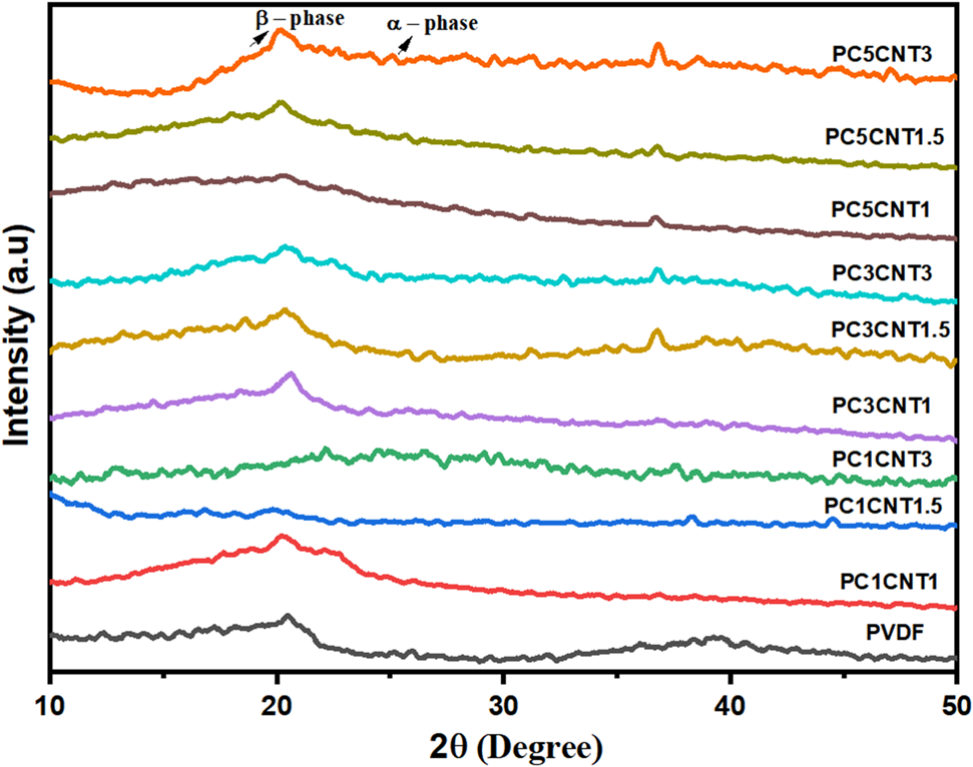
XRD patterns of PVDF and its nanocomposites with Co_3_O_4_ nanowires and multiwall carbon nanotubes (MWCNTs).

The crystallinity of PVDF nanocomposites were evaluated using the PXRD pattern to see the combined impact of hybrid nanomaterial containing Co_3_O_4_–MWCNTover the crystallinity of PVDF films. The PVDF nanocomposite membranes have different content of hybrid Co_3_O_4_–MWCNTs nanostructures as listed in Table 1. Fig. 3 shows the XRD patterns of the pure PVDF and Co_3_O_4_–MWCNTs/PVDF nanocomposite membranes in the range of 5°–80° 2*θ*. XRD graphs of pure PVDF shown two absorption bands located at 2*θ* of 20.5° (100) and 39.45° (211), respectively. The peak at 20.5° showed the alpha phase of PVDF while the absorption peak at 2*θ* of 39.45° refers to the gamma phase of PVDF.^50^ After the addition of Co_3_O_4_–MWCNTs nanostructures with different weight percentages (wt%), the nanocomposites show different peaks from the pure PVDF. The diffractograms of the resulting nanocomposite membranes shown two absorption peaks, one peak exists at 20.5° with less intense and other peak exist at 39.45° of 2*θ* which nearly vanished in all samples, while the peak for β-phase showed higher intensity as compared to pure PVDF. This shown that upon addition of Co_3_O_4_–MWCNTs, a significant result regarding conversion of α- and γ-into β-phase PVDFs occurred.

The XRD pattern of PC1CNT1 nanocomposite showed only one peak at position 20.2° (110) showing little deviation from the peak of pure PVDF, which exist at 20.5° degree showing reflection for (100). The XRD pattern of PC1CNT1.5 nanocomposites, the main peak at 2*θ* position of 20.5° in case of pure PVDF has greatly reduced and two additional bands appeared at 2*θ* position of 7.60°, which is of high intensity and 16.45° is of low intensity. The new peaks appeared because adding nano-fillers in the resulting nanocomposites causes the corresponding β-phase formation. Furthermore, the peak intensity for α-phase PVDF decreases continuously with respect to increasing concentration of Co_3_O_4_–MWCNTs nanostructures in the nanocomposites (Fig. 3). In the case of PC1CNT3 nanocomposite, the XRD pattern showing three low intensities peaks at 2*θ* of 13.2°, 22.27° and 37.40° respectively. Similarly, in case of PC3CNT1, the XRD diffraction showed three peaks at 2*θ* position of 20.3°, 25.9° and 36.7°. The new peaks appeared in the case of PC1CNT3 and PC3CNT1 nanocomposites showing strong interfacial interaction between nano-fillers and PVDF polymer, corresponding to the formation of active polar β-phase PVDF. Furthermore, the XRD pattern of PC3CNT1.5 and PC3CNT3 nanocomposites showed two peaks appear at 20.3° and 36.7° of 2*θ*. The peak at 2*θ* of 20.3° is of high intensity while peak at 36.7° is of less intensity. Furthermore, in case of PC5CNT1 nanocomposite, three peaks appeared in the XRD pattern at 2*θ* position 16.3°, 20.3° and 36.7° of low intensity, followed by PC5CNT1.5 and PC5CNT3 nanocomposites, where two same and distinctive peaks appear at 2*θ* position of 20.3° and 36.7° respectively. The reflection plane (110/200) in the XRD analysis of the resulting nanocomposites of PVDF which are corresponding to the polar β-phase PVDF. From the XRD data, it can be concluded that upon addition of nano-filler, there occurs transformation of nonpolar α into polar β-phase PVDF in the nanocomposites, crystallinity of the resulting nanocomposites also improved.^51^

### 3.3 FTIR analysis

**Fig. 4:**
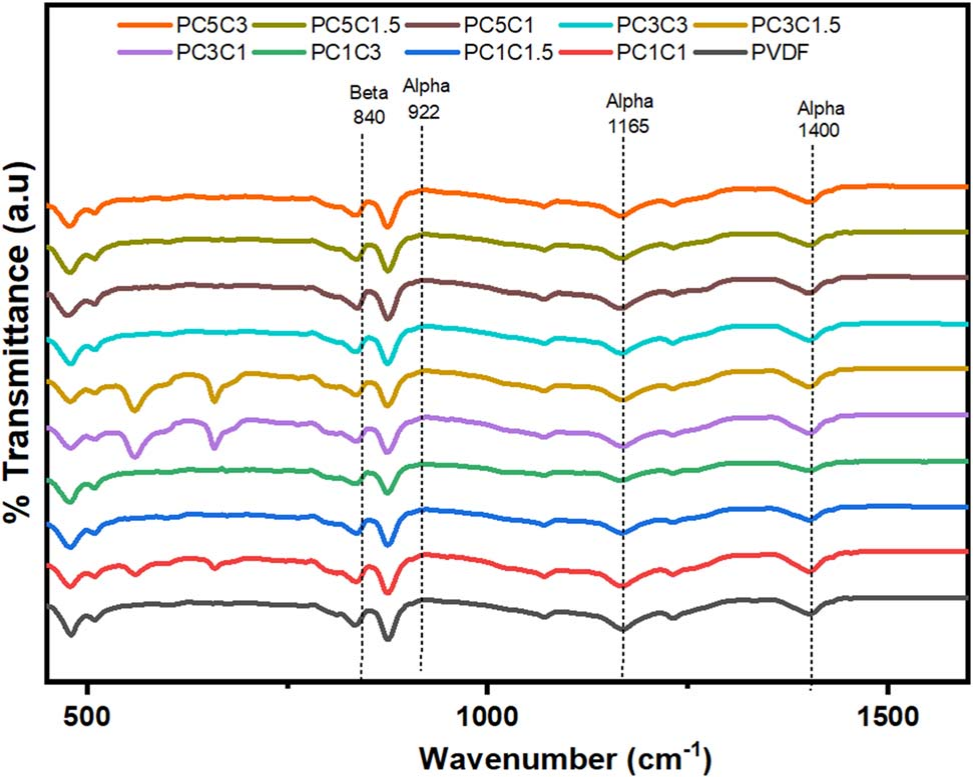
FTIR curves of blank PVDF and PVDF nanocomposites with different wt% of Co_3_O_4_ and functionalized MWCNTs nanostructures.

FTIR analysis of the PVDF nanocomposites with hybrid nanostructures was carried out to see the impact of the nano-filler and co-filler on the crystallinity and morphology of the pure PVDF hybrid nanocomposite membranes. The FTIR spectra of blank PVDF and its nanocomposites loaded with various wt% hybrid nanostructures of Co_3_O_4_–MWCNTs as described in Fig. 4. The FTIR spectrum of pure PVDF showed different absorption bands allocated at position 479 cm^−1^, 515 cm^−1^, 600 cm^−1^, 840 cm^−1^, 876 cm^−1^, 1166 cm^−1^, and 1400 cm^−1^ respectively. These are corresponding to the literature reported elsewhere.^52^ However, in our case upon the loading of Co_3_O_4_–MWCNTs nanostructures, there occurs transformation of nonpolar α into more active polar β-phase PVDF and the peaks for nonpolar α and weak polar γ-phase PVDF appears to be weakened or disappeared, and some additional peaks appeared at position of 563 cm^−1^, 660 cm^−1^, 1275 cm^−1^ respectively. The appearance of new peaks corresponds to the development of β-phase PVDF.^53^ The results obtained from FTIR data showed good agreement with the XRD results. The crystal behavior of PVDF polymer was improved upon the loading of Co_3_O_4_–MWCNTs in all nanocomposites. The FTIR results shown that the peaks intensities for β-phase PVDF was found to be increased with enhanced concentration of Co_3_O_4_–MWCNTs nanostructures in the resulting composites. Furthermore, by increasing the concentration of Co_3_O_4_–MWCNTs, the polar β-phase formation was also increased of the PVDF nanocomposites.

### 3.4 Thermal analysis of the PVDF/Co_3_O_4_–MWCNTs membranes

**Fig. 5:**
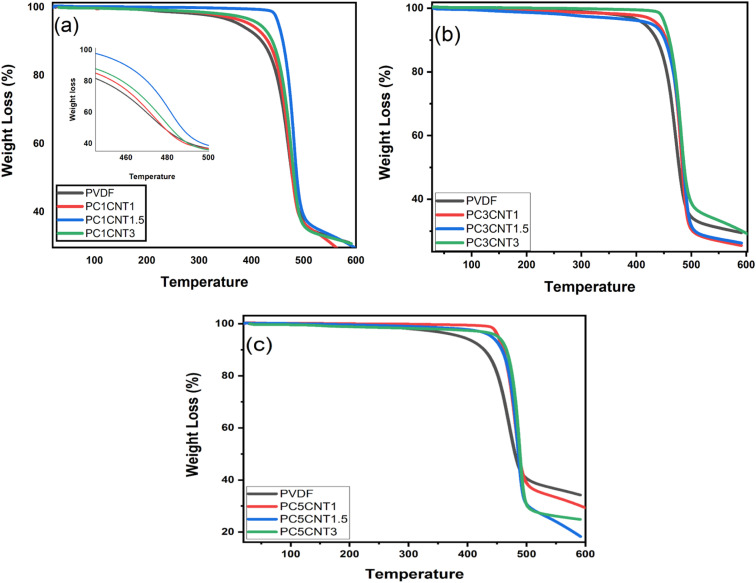
TGA patterns of pure PVDF against its nanocomposite films loaded with different wt% of Co_3_O_4_ and functionalized MWCNTs nanostructures. (a) PVDF, PC1CNT1, PC1CNT1.5, PC1CNT3 (b) PVDF, PC3CNT1, PC3CNT1.5, PC3CNT3 (c) PVDF, PC5CNT1, PC5CNT1.5 and PC5CNT3, respectively.

The thermal analysis of PVDF nanocomposites with Co_3_O_4_–MWCNTs nanostructures was carried out in order to see the effect of the hybrid Co_3_O_4_–MWCNTs materials on the thermal stability and thermal decomposition of the resulting nanocomposite films. The films were heated at constant rate of 10 °C min^−1^ in the range of 25–600 °C. The thermograms of the pure PVDF and PVDF/Co_3_O_4_–MWCNTs nanocomposites presented in Fig. 5 showed that pure PVDF has shown one step degradation after 340 °C which showed that PVDF is stable up to 340 °C. The total weight loss calculated is 66%. The thermal behavior of the films was also studied displaying one step degradation same as pure PVDF. However, a significant variation was found in the thermal behavior of PVDF/Co_3_O_4_–MWCNTs nanocomposites in comparison to pure PVDF. The TGA results showed that the onset temperature (*T*_onset_) and ending temperature (*T*_end_) of the nanocomposites are higher than pure PVDF in all the resulting nanocomposite films. The thermograms shown that thermal stability of PVDF increases continuously upon the addition of Co_3_O_4_–MWCNTs nanostructures. A significant increase was observed in thermal behavior of the PVDF polymer upon the addition of nano-fillers reported previously in the literature.^49–52^ The thermal stability of the prepared nanocomposite membranes increases with enhancing concentration of nano-fillers, which are presented in Fig. 5(a–c). The *T*_onset_ was increased to 401 °C (PC1CNT1), 440 °C (PC1CNT1.5), 449 °C (PC1CNT3), 421 °C (PC3CNT1), 443 °C (PC3CNT1.5), 450 °C (PC3CNT3), 440 °C (PC5CNT1), 423 °C (PC5CNT1.5) and 452 °C (PC5CNT3) upon formation of PVDF/Co_3_O_4_–MWCNTs nanocomposite films with different concentration of Co_3_O_4_–MWCNTs nanostructures, respectively. This shift in the onset of the degradation process towards higher temperature due to the use of higher wt% of nano-fillers could be attributed to the strong attraction of the PVDF chains with the nano-fillers. The reason for the improvement in the thermal stability *versus* thermal decomposition could be due to increased concentration of nanocomposites which avoid the escaping of the degraded product during heating which limiting the continuous degradation of the PVDF. However, in this case, the strong interfacial attraction between the PVDF and Co_3_O_4_–MWCNTs nanostructures may be responsible for withdrawing the heating. The TGA pattern shows that increase wt% of the hybrid nano-fillers (Co_3_O_4_–MWCNTs) increases both the *T*_onset_ and *T*_end_.

**Fig. 6:**
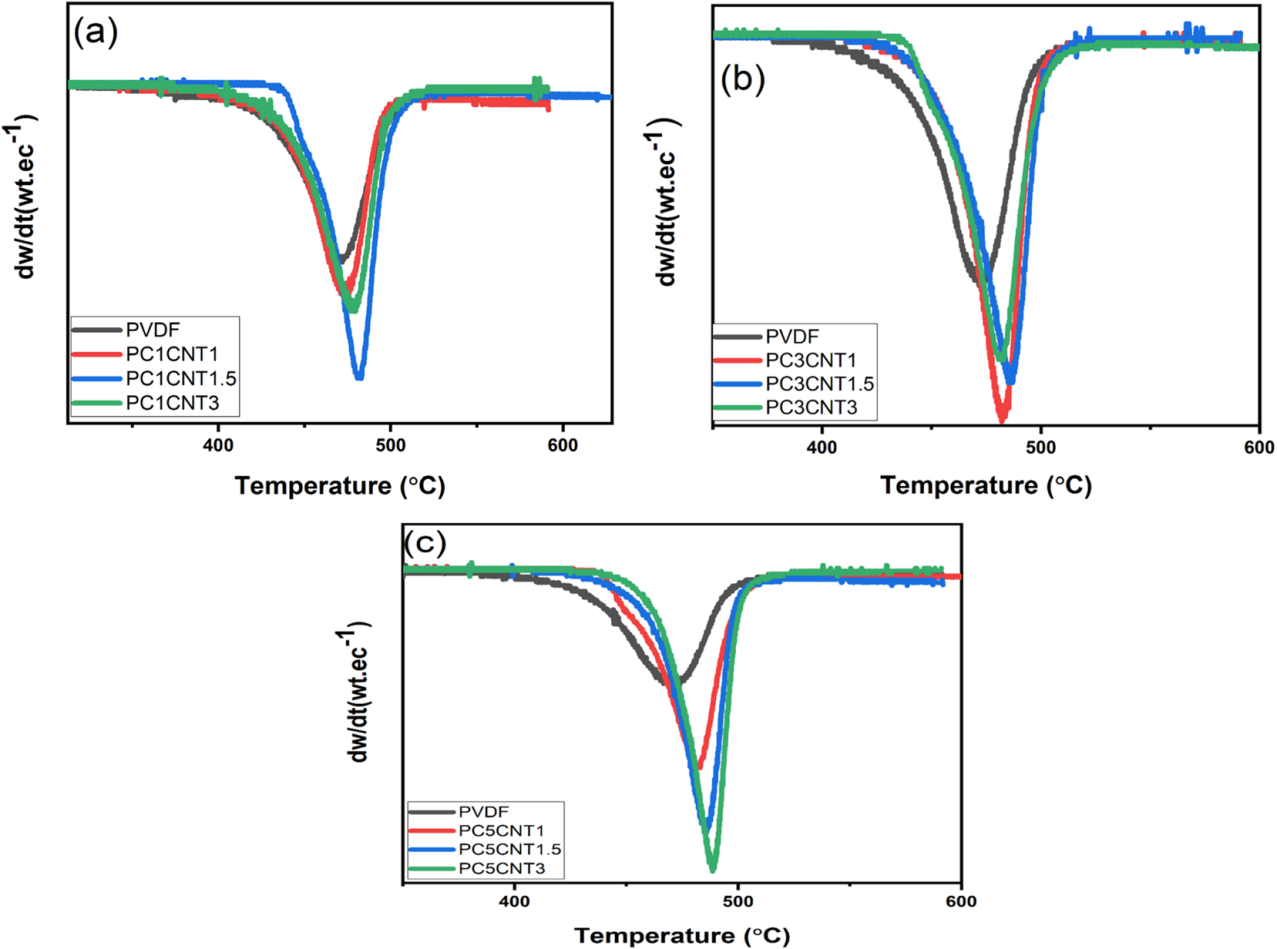
TDA patterns of pure PVDF against its nanocomposite films loaded with different wt% of Co_3_O_4_ and functionalized MWCNTs nanostructures. (a) PVDF, PC1CNT1, PC1CNT1.5, PC1CNT3 (b) PVDF, PC3CNT1, PC3CNT1.5, PC3CNT3 (c) PVDF, PC5CNT1, PC5CNT1.5 and PC5CNT3, respectively.

The differential thermal analysis (DTA) data of the resulting nanocomposites have been abstracted from the TGA data, which are presented in Fig. 6(a–c). The peak temperature (*T*_p_) for all the nanocomposite films were evaluated and found that the value of *T*_p_ increases continuously upon the addition and concentration of fillers (Co_3_O_4_) and co-fillers (MWCNTs)in different wt%.

**Fig. 7:**
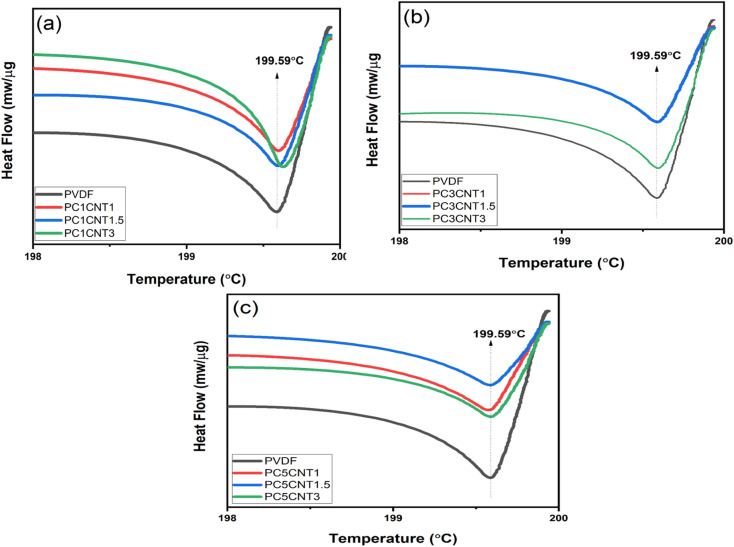
DSC curves of pure PVDF against its nanocomposite films loaded with different wt% of Co_3_O_4_ and functionalized MWCNTs nanostructures. (a) PVDF, PC1CNT1, PC1CNT1.5, PC1CNT3 (b) PVDF, PC3CNT1, PC3CNT1.5, PC3CNT3 (c) PVDF, PC5CNT1, PC5CNT1.5 and PC5CNT3, respectively.

Furthermore, the differential scanning calorimetry (DSC) of the PVDF nanocomposites with Co_3_O_4_–MWCNTs were performed in temperature range up to 200 °C at constant heating rate of 20 °C min^−1^. The purpose of this study was to study the effect of the loading Co_3_O_4_–MWCNTs nanostructures on the phase change of PVDF in its nanocomposites. The melting temperature (*T*_m_) of PVDF in the resulting nanocomposites increases upon the incorporation and concentration of fillers (Co_3_O_4_) and co-fillers (MWCNTs) as compared to pure PVDF as presented in Fig. 7(a–c). However, the increase in *T*_m_ of the prepared films is very small and less significant but these increase in *T*_m_ occurred because of loading and concentration of the fillers in various weights. Addition of Co_3_O_4_–MWCNTs, a significant effect regarding conversion of PVDF phases is resultant to the phase change from non-polar α-into technologically important β-phase PVDF. This can also be achieved by better dispersion of fillers in the polymer matrix.^53,54^ The DSC graphs show a clear trend in the case of higher concentration of fillers.

### 3.5 Electrical properties of PVDF nanocomposite films

**Fig. 8:**
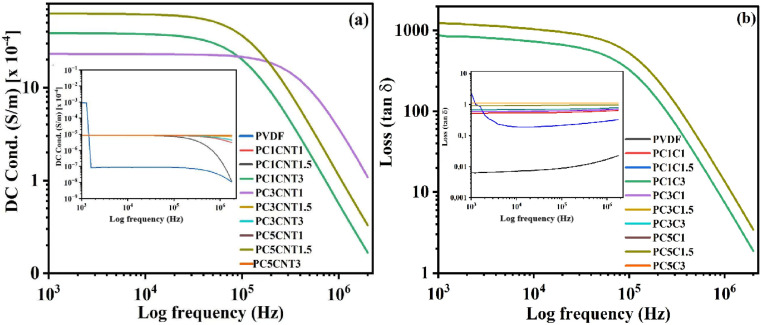
DC conductance (a) and dielectric loss (b) of blank PVDF and its nanocomposites loaded with different wt% of Co_3_O_4_ and functionalized MWCNTs nanostructures.

DC conductance of the synthesized PVDF/Co_3_O_4_–MWCNTs nanocomposites were studied and measured using an impedance spectroscopy. The pertinent data of the electrical properties are given in Fig. 8(a and b). The DC conductance of the synthesized PVDF nanocomposite films were measured in the frequency range 1 × 10^3^ Hz to 1 × 10^6^ Hz at room temperature. The DC conductance shown by pure PVDF is very low, which decreases by increasing the frequency (Fig. 8(a)). Similarly, the DC conductance of the synthesized PVDF nanocomposites loaded with hybrid nano-fillers (Co_3_O_4_–MWCNTs) in different wt% showed a good result in most cases. All the samples showed higher DC conductance as compared to pure PVDF which is initially increasing very quickly with enhancing frequency and then decreased with increasing frequency.^55^ The DC conductance of synthesized nanocomposites with Co_3_O_4_–MWCNTs hybrid fillers depend on both the concentration and distribution of these fillers within the matrix. In samples with higher filler loadings, the conductive network formed by the Co_3_O_4_ and MWCNTs facilitating electron mobility through a continuous conductive pathway. This network formation allowed electrons to hop between conductive particles, significantly enhancing the overall conductivity of the composite. In contrast, samples with lower filler concentrations, the conductive fillers are not close enough to form a continuous path, resulting in poor connectivity between conductive regions and a low probability for electron hopping. Additionally, the distribution and possible aggregation of fillers in these samples could contribute to the observed minimal changes in conductivity, as non-uniform dispersion can inhibit the formation of an effective conductive network. Similarly, the higher DC conductance of PVDF nanocomposites in case of more contents of fillers showed direct relation between DC conductance and concentration of fillers in the synthesized films. The higher conductance values could be attributed to the strong interaction between fillers and PVDF on one hand, and on the other hand, increasing the formation of polar β-phase of PVDF in its nanocomposites.^56^ Similarly, a noticeable increase in conductance was observed with the addition of Co_3_O_4_–MWCNTs, suggesting that the hybrid nanostructures facilitated charge transfer within the composite matrix. However, it is important to note that part of the increased conductance may be attributed to electrode effects, particularly the interaction between the composite material and the electrode surfaces during measurement.

Similarly, the dielectric loss of the synthesized films was measured at room temperature (Fig. 8(b)). The dielectric loss of pure PVDF and its nanocomposites with Co_3_O_4_–MWCNTs were plotted as a function of frequency, depicted in Fig. 8(b). The resultant loss of blank PVDF and the prepared films constantly decreases with enhancing frequency (Hz). There was observed no strong fluctuation in the loss of the films. The reason for this might be the Maxwell–Wagner–Siller (MWS) polarization effect, due to strong polarization on the surface of PVDF polymer upon fillers incorporation.

## 4 Conclusions

The present study proves the successful transformation of the non-polar α-phase of PVDF into the technologically important β-phase by reinforcing the polymer with 1D hybrid Co_3_O_4_–MWCNTs nanostructures. The Co_3_O_4_ nanowires were synthesized through an electrospinning technique, while the functionalized MWCNTs were used to improve distribution and interaction with the PVDF matrix. The resulting PVDF/Co_3_O_4_–MWCNTs nanocomposites were characterized using various techniques, including XRD, FTIR, TGA, TDA, DSC, and impedance spectroscopy. XRD analysis confirmed the improvement in crystallinity, and FTIR spectroscopy verified the presence of the polar β-phase PVDF. Thermal analyses (TGA, TDA, and DSC) showed that the addition of Co_3_O_4_–MWCNTs significantly enhanced the thermal stability of the nanocomposites, with an increase in both thermal resistance and decomposition temperatures. While the findings of this study contribute to the development of high-performance PVDF nanocomposites, several limitations should be acknowledged. The transformation efficiency from the α-phase to the β-phase could vary depending on the concentration and morphology of the nanostructures, which permits further optimization.

For future studies perspectives, it is recommended to investigate the effects of varying the ratio of Co_3_O_4_ to MWCNTs, as well as the influence of different hybrid nanostructures on the phase transformation and mechanical properties of the composites. Moreover, exploring the long-term stability and practical applications of these PVDF/Co_3_O_4_–MWCNTs nanocomposites in real-world devices, such as sensors and actuators, will provide deeper insights into their potential for industrial use. Further work should also focus on scalability and cost-effectiveness in fabricating large-scale nanocomposite films, to evaluate their commercial viability. Due to enhanced thermal and electrical properties, the synthesized membranes could be used in many electronic devices such as sensors and capacitors.

## Data availability

The data that support the findings of this study are available from the corresponding author upon reasonable request. Due to [state any restrictions, *e.g.*, privacy or ethical concerns], some data may not be publicly available.

## Conflicts of interest

The authors declare no conflict of interest.
